# Exposure to psychosocial work strain and changes in smoking behavior during pregnancy – a longitudinal study within the Danish National Birth Cohort

**DOI:** 10.5271/sjweh.3921

**Published:** 2020-12-16

**Authors:** Kristina Mattsson, Karin Sørig Hougaard, Camilla Sandal Sejbaek

**Affiliations:** 1 Division of Occupational and Environmental Medicine, Institute of Laboratory Medicine, Lund University, Lund, Sweden; 2 Reproductive Medicine Center, Skåne University Hospital, Malmö, Sweden; 3 National Research Centre for the Working Environment, Copenhagen, Denmark; 4 Department of Public Health, University of Copenhagen, Copenhagen, Denmark

**Keywords:** Denmark, job strain, pregnant population, psychosocial stress, smoking cessation

## Abstract

**Objective:**

Knowledge of the relationship between psychosocial strain in the work environment and smoking during pregnancy is scarce. This study aimed to examine the association between psychosocial job strain and change in smoking behavior during pregnancy.

**Methods:**

The cohort included 65 645 pregnancies from the Danish National Birth Cohort (1996–2002), where pregnant women were interviewed on job factors and lifestyle during the first and third trimesters. Smoking was categorized into non-, non-daily, and daily smoking at each interview. Psychosocial job strain was categorized into four groups based on the concept of Karasek’s demand–control model: low strain (reference), passive, active and high strain. Associations between psychosocial strain and change in smoking status between the first and second interviews were analyzed by multinomial logistic regression, separately for each smoking category at first interview.

**Results:**

Non-smoking women exposed to high strain work were more likely to become daily smokers [adjusted odds ratio (OR_adj_) 1.41, (95% confidence interval (CI) 1.08–1.83)] compared to non-smoking women exposed to low strain work. Non-smoking women exposed to passive work were more likely to become both non-daily and daily smokers [OR_adj_ 1.59 (95% CI 1.21–2.08) and OR_adj_ 1.32 (95% CI 1.03–1.70), respectively]. Daily smoking women exposed to high strain work were less likely to decrease their smoking [OR_adj_ 0.57 (95% CI 0.32–0.99)] compared to daily smoking women exposed to low strain work.

**Conclusions:**

Psychosocial strain influenced the women’s smoking behavior during pregnancy, especially in job types with low control.

It is well established that smoking during pregnancy is detrimental for perinatal health; yet smoking is still common among pregnant women in several countries (1). In Denmark, around 9% of all pregnant women smoke during early pregnancy and almost 7% continue throughout the pregnancy (2). To help women quit smoking during pregnancy it is important to understand which factors influence smoking behavior during pregnancy.

Studies among male and non-pregnant female smokers indicate that work conditions, such as stress, are associated with smoking intensity, probability of cessation and the risk of relapse (3–5). High psychosocial strain at work could contribute to continuous smoking in several ways. Smoking itself might represent a way of coping with high demands in the work situation; its stress-relieving properties are often stated as a reason for continued smoking (6, 7). Further, experience of high strain at work might leave little psychological room for undertaking another challenge such a smoking cessation (7).

A majority of women of reproductive age are occupationally active (76%, Denmark, 2015) (8) and spend a large part of their time at work, also during pregnancy (9). Earlier results from a non-pregnant population on the influence of psychosocial work strain might not be generalizable to a pregnant population. Pregnant women conceivably differ in their motivation to quit smoking compared to their non-pregnant counterparts (10, 11). At the same time they might face a different setting of stressors, and thus, pregnancy might not be seen as a possible time to cope with smoking cessation (7). Additionally, the societal pressure and focus on the need to quit smoking could actually make it harder to do so (11).

Earlier research examining work-related psychosocial strain has reported that high strain is associated with continued smoking during pregnancy, however, the studies are few (12–15), were done in small populations (13, 14), used a cross-sectional design (12), or did not distinguish work-related strain from other stressful life events (12–15). Lastly, none of the studies examined associations between work strain and smaller changes in smoking intensity, such as a reduction in the number of cigarettes smoked. The only two studies that explicitly investigated psychosocial job strain focused on the timeframe between conception and up until the end of the first trimester (14, 15). No studies have investigated potential associations after the first trimester. Studying maternal smoking behavior for this period of gestation is important as women who quit smoking before the third trimester show the same risk of low birth weight and preterm birth as women who never smoked during pregnancy (16–20).

In the present study, using a large, previously established pregnancy cohort – the Danish National Birth Cohort (DNBC), we aimed to investigate if psychosocial strain at work influenced smoking status between the first and the third trimester. We also investigated if the likelihood of change depended on the women being a non-, non-daily, or daily smoker in the first trimester. We hypothesized that women experiencing high psychosocial strain at work were more likely to continue smoking or to increase their level of smoking in comparison to women experiencing low strain at work.

## Methods

### Study population

The study population was the DNBC, a nation-wide, population-based cohort consisting of 101 042 pregnancies, enrolled in 1996–2002 (21). The pregnant women were recruited by their general practitioner at their first antenatal visit. In Denmark, this normally takes place during gestational weeks 6–12, and almost all pregnant women undergo maternal healthcare (22). Approximately 50% of the general practitioners participated, and about 60% of the invited pregnant women participated in the DNBC (21). To become a part of this cohort, the women should be pregnant, intend to carry the pregnancy to term, live in Denmark, and be able to participate in a telephone interview in Danish. Two interviews were conducted during pregnancy – at early (12–14 weeks) and late (30–32 weeks) pregnancy. The first interview included topics such as maternal health habits, medical problems, and medication as well as physical and psychosocial working environment. The second interview followed up many of these topics.

This study included data from both interviews in which a total of 82 646 women participated. For this study, the women should be pregnant at both interviews, be working, and have valid data on smoking behavior and psychosocial job strain exposure (N=67 408). Unemployed women were not included as the questionnaire only addressed demands and control at work. Women with missing data on any of the covariates included in the subsequent analyses were excluded. The final study population included 65 646 pregnancies, with complete information on all relevant variables.

Data was pseudo-anonymized before they were accessed via Statistics Denmark. Permissions to use and store data were obtained from the DNBC and the Danish Data Protection Agency. Danish legislation requires approval from the Ethical Committee only for use of human tissue; hence, no ethical approval was needed.

### Outcome – change in smoking behavior during pregnancy

The main outcome of interest was change in smoking behavior between the first and the second interview. We additionally investigated change in smoking prior to the first interview. There were three questions on smoking behavior in the first interview: “Did you smoke at any time during your pregnancy?”, “Are you smoking right now?”, and “Have you smoked at any time during pregnancy, including very first time after conception?”. In the second interview, the questions were: “Have you been smoking since the last interview?” and “Are you smoking right now?”. An affirmative answer to these questions led to a follow-up question where the participant was asked to quantify the number of cigarettes smoked and if the smoking was daily or non-daily. The information on number of cigarettes was not usable for this study due to the low quality of the data. Smoking during pregnancy was therefore categorized at both the first and second interviews as follows: (i) non-smoking, (ii) non-daily smoking, and (iii) daily smoking. For each of these smoking strata at the first interview, the women could either maintain their smoking level, decrease or increase their levels until the second interview.

### Exposure - Psychosocial strain at work

**Figure 1:**
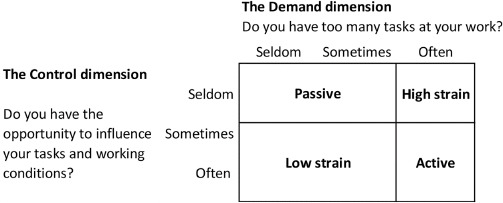
Categories of exposure to psychosocial strain according to the answers in the Danish National Birth Cohort. Figure originally by Larsen et al (36).

The women’s psychosocial working environment was estimated according to the concept of Karasek’s demand–control model (23). Information on the demand and control dimensions of the model was extrapolated from the first interview in the DNBC based on the questions: “Do you have too many tasks at your work?” (denoting the demand dimension) and “Do you have the possibility to influence your work tasks and working conditions?” (denoting the control dimension). The questions could be answered with seldom, sometimes, and often. The questions were combined according to these responses into four categories of psychosocial strain: (i) low (low demands, high control), which served as the reference category, (ii) passive (low demands, low control), (iii) active (high demands, high control) and lastly (iv) high (high demands, low control) strain (23). Figure 1 depicts how the categorization was done according to the answers listed above.

### Covariates

The following covariates were decided a priori and retrieved from the first DNBC interview: maternal age at conception (categorized in the models: <25, 25–29, 30–34, and ≥35 years), maternal body mass index (BMI) before pregnancy (<18.5, 18.5–<25, 25–<30, and ≥30 kg/m^2^), parity/number of previous children (0, 1 and ≥2), socio-economic position derived from self-reported job titles (high educational level, medium educational level, skilled work, unskilled work, student), exposure to second-hand smoke/partner smoking (no exposure, non-daily exposure, daily exposure), and exposure to passive smoking (in this case partner’s smoking). We chose to included partner’s smoking rather than cohabiting status as this was considered to have a larger influence on smoking habits.

### Statistical analyses

The associations between psychosocial job strain and smoking behavior between the first and the second interview were explored using multinomial logistic regressions generating odds ratios (OR) and 95% confidence intervals (CI), to account for the three possible outcomes (no change, decrease or increase in smoking level). The three strata of smoking status at the first interview (non-smoking, non-daily smoking and daily smoking) were analyzed separately, with no change in smoking habit considered the reference category. In all analyses, the different categories of psychosocial strain were compared to low strain. All analyses, including the crude estimates, were analyzed with a cluster term to account for dependency, since some of the women contributed with more than one pregnancy in the cohort (N=3483).

Initial models investigated job strain separately, after which we included the covariates described above.

To investigate the robustness of the results, we also performed the following subgroup analyses to investigate if patterns in change in smoking behavior differed between the following groups: (i) stratifying primiparous from multiparous women and (ii) stratifying socio-economic position into high/medium education and skilled/unskilled work (excluding students). Lastly, we also investigated the odds of quitting smoking at any time before the second interview, among the women having smoked at any time during pregnancy (including both the period prior to the first interview and between the first and second interviews). Conventional logistic regression analyses were used for these analyses as the women were only asked whether or not they had been smoking, without specification of the amount smoked. All analyses were carried out in Stata version 13.1 (StataCorp, College Station, TX, USA).

## Results

There were 15 900 women (24.2%) stating that they had smoked during pregnancy prior to the first interview. The daily smoking prevalence was 13.6% and 13.9% at the first and second interview, respectively. Fewer women stated that they were non-daily smokers (1.5% and 1.4%, respectively). Thus, the rate of any pregnancy smoking (both daily and non-daily) was reduced from 24.2% to 15.1% by the time of the first interview. Table 1 shows the baseline characteristics among the included women, stratified on smoking status at the first interview. Daily smokers were younger, had lower educational levels and were more often exposed to daily secondhand smoke by their partner, compared to non-smokers (table 1).

**Table 1:** Characteristics of women included in study population by smoking status at 1^st^ interview. [BMI=body mass index.]

Women (N=65 646)	Non-smokers (N=55 760)	Non-daily smokers (N=962)	Smokers (N=8924)
		
	N (%)	N (%)	N (%)
Maternal age at conception (yrs)			
<25	5356 (9.6)	145 (15.1)	1466 (16.4)
25–29	23 505 (42.2)	397 (41.3)	3346 (37.5)
30–34	20 379 (46.6)	315 (32.7)	2944 (33.0)
≥35	6520 (11.7)	105 (10.9)	1168 (13.1)
Pre-pregnancy BMI (kg/m^2^)			
<18.5	2058 (3.7)	45 (4.7)	604 (6.8)
18.5–24.9	38 424 (68.9)	700 (72.8)	5755 (64.5)
25–29.9	10 893 (19.5)	159 (16.5)	1817 (20.4)
≥30	4385 (7.9)	58 (6.0)	748 (8.4)
Parity/previous children			
0	26 540 (47.6)	519 (54.0)	3965 (44.4)
1	20 859 (37.4)	306 (31.8)	3350 (37.5)
≥2	8361 (15.0)	137 (14.2)	1609 (18.0)
Socio-economic position			
High educational level	6758 (12.1)	75 (7.8)	426 (4.8)
Medium educational level	19 556 (35.1)	283 (29.4)	1924 (21.6)
Skilled work	12 287 (22.0)	210 (21.8)	1846 (20.7)
Unskilled work	13 838 (24.8)	330 (34.3)	4206 (47.1)
Student	3321 (6.0)	64 (6.7)	522 (5.9)
Exposed to second-hand smoke^a^			
No	42 582 (76.4)	484 (50.3)	3357 (37.6)
Non-daily	1658 (3.0)	82 (8.5)	154 (1.7)
Daily	11 520 (20.7)	396 (41.2)	5413 (60.7)
Work strain b			
Low strain	33 898 (60.8)	550 (57.2)	4582 (51.3)
Passive work	4970 (8.9)	96 (10.0)	1109 (12.4)
Active work	12 646 (22.7)	227 (23.6)	2122 (23.8)
High strain	4246 (7.6)	89 (9.3)	1111 (12.5)

a Partner’s smoking.

b Based on the demand–control model: low demand + low control = passive work; high demands + high control = active work; high demands + low control = high strain; low demands + high control = low strain.

Table 2 shows changes in smoking status between the first and second interview. The percentage of women who changed their smoking behavior was highest among the non-daily smokers, where 76.2% changed their smoking behavior until the second interview compared to 2.0% and 9.2% among non-smokers and daily smokers, respectively.

**Table 2:** Change in smoking status between the first and second interview (N and %), relative to smoking strata in the first interview.

Change in smoking behavior between 1^st^ and 2^nd^ interview	N (%)
Non-smoking at 1^st^ interview	
Still non-smoking (no change)	54 652 (98.0)
Increase to non-daily smoking	511 (0.9)
Increase to daily smoking	597 (1.1)
Non-daily smoking at 1^st^ interview	
Still non-daily smoking (no change)	229 (23.8)
Increase to daily smoking	417 (43.4)
Decrease to non-smoking	316 (32.9)
Daily smoking at 1^st ^interview	
Still daily smoking (no change)	8100 (90.8)
Decrease to non-daily smoking	172 (1.9)
Decrease to non-smoking	652 (7.3)

For women reporting any smoking during pregnancy (also before the first interview), the crude and adjusted OR (OR_adj_) of quitting at any time before the second interview are shown in table 3. Compared to the reference category (low strain), women experiencing high strain were less likely to quit smoking [OR_adj_ 0.87 (95% CI 0.78–0.98)].

**Table 3:** Crude and adjusted odds ratios (OR_adj_) of quitting at any time in pregnancy (ie, non-smoker 2^nd^ interview2) provided reporting of any smoking during pregnancy in the 1^st^ and the 2^nd^ interview (including the time before the first interview). [CI=confidence interval.]

N=15 900	Odds of quitting at any time before 2^nd^ interview	

	Crude OR (95% CI)	OR_adj_^a^ (95% CI)
Job strain		
Low	Reference	Reference
Passive	0.78 (0.70–0.86)	0.89 (0.80–1.00)
Active	0.95 (0.88–1.03)	0.94 (0.87–1.02)
High	0.75 (0.67–0.84)	0.87 (0.78–0.98)

a Adjusted for maternal age at conception, body mass index, parity, socio-economic position and exposure to secondhand-smoke.

Table 4 shows the crude and OR_adj_ following multinomial logistic regression analyses performed separately for each of the three smoking strata at interview 1. Non-smoking women experiencing high strain were more likely to become daily smokers [OR_adj_ 1.41 (95% CI 1.08–1.83)] compared to non-smoking women experiencing low strain; and non-smoking women in the passive group were more likely to become both non-daily and daily smokers [OR_adj_ 1.59 (95% CI 1.21–2.08) and OR_adj_ 1.32 (95% CI 1.03–1.70), respectively].

**Table 4:** Crude and adjusted odds ratios (OR_adj_) and 95% confidence intervals (CI) for change in smoking behavior between the 1^st^ and the 2^nd^ interview by job strain. Women that were non-smoking, non-daily smoking and daily smoking at the 1^st^ interview were analyzed separately. The reference category (ref) in each group is the maintained smoking behavior, ie, no change in smoking status.

Smoking status	N (%)	Low strain work	Passive work		Active work		High strain work	
		
			Crude OR (95% CI)	OR_adj_^a^ (95% CI)	Crude OR (95% CI)	OR_adj_^a^(95% CI)	Crude OR (95% CI)	OR_adj_^a^(95% CI)
Non-smoking	55 760 (84.9)							
Increase to non-daily smoking	511 (0.9)	ref	1.65 (1.26–2.16)	1.59 (1.21–2.08)	1.22 (0.99–1.50)	1.22 (0.99–1.51)	1.06 (0.75–1.50)	1.03 (0.73–1.46)
Increase to daily smoking	597 (1.1)	ref	1.60 (1.25–2.06)	1.32 (1.03–1.70)	0.97 (0.79–1.20)	0.98 (0.80–1.21)	1.68 (1.29–2.18)	1.41 (1.08–1.83)
Non-daily smoking	962 (1.5)							
Increase to daily smoking	417 (43.4)	ref	1.10 (0.62–1.93)	0.96 (0.53–1.73)	1.49 (0.99–2.23)	1.42 (0.94–2.16)	1.21 (0.71–2.07)	1.09 (0.62–1.89)
Decrease to non-smoking	316 (32.9)	ref	1.16 (0.65–2.07)	1.15 (0.64–2.07)	1.20 (0.78–1.85)	1.18 (0.76–1.83)	0.55 (0.29–1.05)	0.53 (0.27–1.03)
Daily smoking	8924 (13.6)							
Decrease to non-daily smoking	172 (1.9)	ref	0.61 (0.36–1.04)	0.67 (0.39–1.14)	0.70 (0.48–1.03)	0.67 (0.45–0.98)	0.53 (0.30–0.93)	0.57 (0.32–0.99)
Decrease to non-smoking	652 (7.3)	ref	0.94 (0.73–1.21)	1.01 (0.78–1.31)	1.01 (0.84–1.24)	0.99 (0.76–1.29)	0.91 (0.70–1.18)	0.99 (0.74–1.29)

a Adjusted for maternal age at conception, body mass index, parity, socio-economic position and exposure to secondhand-smoke.

Among non-daily smoking women, there was no statistically significant association between psychosocial strain and change in smoking behavior, but there was a trend toward a lower OR for quitting smoking among women exposed to high strain work [OR_adj_ 0.53 (95% CI 0.27–1.03)] compared to non-daily smoking women exposed to low strain.

Women smoking daily at the first interview were less likely to decrease their smoking to non-daily smoking, if they were exposed to high psychosocial strain [OR_adj_ 0.57 (95% CI 0.43–0.99)], compared to women in the low strain group. There were no associations between psychosocial strain and their likelihood to stop completely [OR_adj_ 0.99 (95% CI 0.74–1.29)].

All estimates remained more or less unchanged after adjustment for potential confounders, except for non-smoking women experiencing passive work or high psychosocial strain, respectively, where point estimates were somewhat lower in the adjusted analyses (table 4).

The subgroup analyses did not reveal notable changes to the overall patterns described above, apart from widening of some of the confidence intervals due to smaller numbers of women (data not shown).

## Discussion

In this large national birth cohort, the overall findings indicated that psychosocial strain at the workplace influenced smoking behavior during pregnancy, particularly among the women who experienced low levels of control at work (the passive and high strain categories). Women who did not smoke at the first interview were more likely to increase their level of smoking if they belonged to these two categories of psychosocial strain. High strain at work was furthermore linked to a lower propensity to reduce smoking if the women were daily smokers at the first interview. Our study additionally showed that most women who stopped smoking did so in the early part of pregnancy (the prevalence of any pregnancy smoking reduced from 24.2% to 15.1% before the first interview took place), which is in line with earlier findings (24).

The finding that many women increased their smoking during pregnancy was somewhat unexpected, although there are qualitative findings that support this pattern (7). Compared to the other smoking strata, non-daily smoking women had the largest proportions of change in smoking behavior in either direction (32.9% decreased and 43.4% increased their smoking). This was, however, not statistically significantly related to psychosocial job strain in our analyses, possibly due to the small numbers in this group. It could be speculated that non-daily smoking women are more prone to changing of smoking behavior *before* pregnancy, and therefore are more likely to change behavior also *during* pregnancy. The non-daily smokers could also reflect women who smoked before pregnancy who did not manage to quit completely and, thus, resumed smoking during pregnancy. From a clinical perspective, this group, even though it is small, might be an important target group for additional support during pregnancy. Indeed, increasing worry about the upcoming birth has been reported as a cause of increased smoking in the later stages of pregnancy (7). Also, low levels of support during pregnancy has been shown to be linked with a higher risk for continued smoking, where a Swedish study found instrumental support (ie, access to advice, information and practical service) to be the most important for smoking cessation (14). Similarly, the importance of adequate maternal healthcare for smoking cessation has also been highlighted in a recent review (25). Interestingly, job support was not related to the risk of persistent smoking in the Swedish study cited above (14).

Among the women who reported not smoking at the first interview, 2% increased their level of smoking. This proportion was on par with the proportion of daily smokers who managed to quit (1.9%) from the first to the second interview. Unfortunately, we did not have information on smoking before pregnancy in the DNBC. We hypothesize that the non-smoking women who increased their smoking levels during pregnancy are likely women who had stopped smoking before or very early in pregnancy and then resumed smoking, rather than never-smokers beginning to smoke during pregnancy, even though the latter scenario is a possibility: an American study found that 3.3% of women who had never smoked started smoking when they were pregnant (26). The study, however, was performed in a specific population of low-income urban women with low educational attainment. We could not find any reports on incident pregnancy smoking in a more representative sample or a Nordic population.

Pregnancy is often viewed as a window of opportunity for smoking cessation. Nonetheless, quitting rates vary considerably. The majority of the published studies show that more than half of the women who smoked during pregnancy failed to quit (as reviewed by Schneider et al) (27). Generally, the scientific discussion considers complete quitting and potential smoking relapse post-partum (28), but the present study suggests that the potential risk for increased smoking or relapse *during* pregnancy should be addressed further.

One explanation for this pattern might be that smoking is used as a coping strategy in the handling of increased demands (6, 7). The burden to quit then becomes two-fold: not only does the woman lose a way of coping with stress, but quitting would then add another demand to an already strained situation. Paradoxically, the pressure to stop smoking could then actually reinforce that same habit as smoking is used to cope also with this demand (11, 12).

The finding in our study, that women exposed to high strain are less likely to quit or decrease their smoking, confirms prior research but in a much larger population (14, 15). Previous studies of job strain in relation to smoking during pregnancy are few and most were performed in small populations (12–15). Additionally, these studies only considered major changes in smoking behavior such as odds of persistent smoking (14) quitters vs non-quitters (15) and abstention fraction, i.e. the percentage of non-smokers among pre-pregnancy smokers (12). No previous studies have considered non-daily smoking in their analyses. Exposure classifications varied between studies: two studies used the psychosocial strain model according to Karasek with multi-item measurements denoting the two dimensions (14, 15). A Norwegian study evaluated psychosocial exposure based on several independent questions regarding workload and opportunities to limit it (12). Lastly, an American study applied a composite measure of any type of stress (emotional, financial, work-related) (13).

The present study adds new information on the influence of psychosocial job strain on smoking behavior during pregnancy. We had information on smoking behavior during the third trimester, which is important in order to investigate whether any cessation or reduction in smoking was maintained. We were, furthermore, able to investigate if the likelihood of change depended on the women’s smoking level early in pregnancy. Other strengths of the present study include the longitudinal design of the study, the large population and good generalizability of the results as the women worked in an array of different trades, had different educational levels and demographic characteristics.

The present study also has several limitations. First, all data are based on self-report. Concerns have been risen regarding the validity of self-reported data on smoking during pregnancy, considering that there is a stigma surrounding smoking in this period. This might lead women to underreport their true smoking status. Nonetheless, a study from Sweden comparing self-reported smoking to a biomarker of nicotine exposure indicated that women tend to report their smoking behavior truthfully (29). Thus, we believe that using self-reported data on smoking status is justified.

The job strain model could constitute a weakness of the study, as only one question was used to reflect each of the demand and control dimensions, respectively. We are not aware of any studies that have validated the specific two questions used in our study to capture psychosocial job strain. Potentially, use of different questions might have categorized the women differently, which could have changed the results. On the other hand, a study validating the use of two single-item measures of stress concluded that it had similar validity as the use of multi-item measures (30). We also assumed that job exposure remained constant throughout pregnancy, since data on working conditions were only present in the first interview. These conditions could have changed, particularly among those exposed to high strain, if preventive measures or work adjustments were implemented.

The complex interplay between work-related strain and psychosocial strain in private life also ought to be considered. There were no questions in the first DNBC-interview specifically related to stressors in private life; hence, this could not be accounted for. However, an Australian study investigating the contribution of stress at work and outside work relative to development of common mental disorders found that the effect of work-stressors could not be explained by co-exposure to stressors outside work (31). Also, according to the Danish national questionnaire survey ‘The Danish Work Environment Cohort Study’, among people who report suffering from stress, a much higher frequency report that the stress is work-related rather than related to private life (32).

Another potential limitation is that the data was collected more than 20 years ago. The rate of pregnancy smoking has since decreased from around 23% in 2000 to 9% in 2017 (2). It is more difficult to evaluate changes in the psychosocial work environment. Overall, including stress both at and outside of work, stress seems to have increased (33, 34). However, according to ‘the Danish Work Environment Cohort Study’, indicators of neither demand nor control has changed between 2010 to 2018, indicating that job strain have been relatively constant (32). In summary, since pregnancy smoking is still prevalent, and psychosocial work strain at best levels with the period of data collection, we believe the findings from the present study are still likely to have bearings on today’s working population, especially since there are signs of a rise in smoking in younger age groups (35).

The benefits of smoking cessation during pregnancy have been established multiple times (16–19). Generally, women who quit smoking during the first trimester have the same risk of giving birth pre-term or to a child with low birth weight as a non-smoking woman (16, 20). They also reduce their risk of placenta previa/ablatio, stillbirth and neonatal mortality otherwise associated with smoking during pregnancy (24).

In conclusion, this study indicates that exposure to psychosocial strain at work is associated with a decreased likelihood of reducing smoking during pregnancy, in particular for work types with low control. Further studies with more detailed classification of smoking habits and work task exposures during pregnancy are needed to elucidate the findings. Intervention studies would further contribute to determine if there are benefits, in terms of change in smoking habits, to be gained by an adjustment of the psychosocial work environment for pregnant women.

## Funding

In this study, the costs associated with access to and use of data were covered by a grant from the Danish Work Environment Research Foundation (grant 20150018124/3).

The Danish National Birth Cohort was established with a significant grant from the Danish National Research Foundation. Additional support was obtained from the Danish Regional Committees, the Pharmacy Foundation, the Egmont Foundation, the March of Dimes Birth Defects Foundation, the Health Foundation and other minor grants. The DNBC Biobank has been supported by the Novo Nordisk Foundation and the Lundbeck Foundation. Follow-up of mothers and children have been supported by the Danish Medical Research Council (SSVF 0646, 271-08-0839/06-066023, O602-01042B, 0602-02738B), the Lundbeck Foundation (195/04, R100-A9193), The Innovation Fund Denmark 0603-00294B (09-067124), the Nordea Foundation (02-2013-2014), Aarhus Ideas (AU R9-A959-13-S804), University of Copenhagen Strategic Grant (IFSV 2012), and the Danish Council for Independent Research (DFF – 4183-00594 and DFF - 4183-00152).

## Competing interests

The authors declare that they have no competing interests to disclose. All authors have had full access to the data presented in the study and take responsibility for the integrity of the data and accuracy of the data analysis.
